# Multimorbidity Patterns and Quality of Life of Elderly Individuals Attending the Center for Health and Wellbeing at a Tertiary Care Hospital: An Observational Study

**DOI:** 10.7759/cureus.78646

**Published:** 2025-02-06

**Authors:** Praveen Ganganahalli, Mallikarjun Yadavannavar, Rekha Udgiri

**Affiliations:** 1 Community Medicine, BLDE (Deemed to be University) Shri. B. M. Patil Medical College Hospital and Research Center, Vijayapura, IND

**Keywords:** elderly, multimorbidity pattern, old age, quality of life, wellbeing

## Abstract

Introduction: Although old age is inevitable, it can be delayed and maintained in good health by researching the causes of low quality of life (QOL) and mitigating them through medical intervention or counseling. Both objective and subjective aspects are frequently used to assess QOL.

Objective: This study aims to analyze morbidity patterns and QOL among elderly patients visiting the Centre for Health and Wellbeing, a tertiary care hospital.

Methodology: An observational study was conducted on elderly patients over 60 undergoing general health screenings at the Department of Community Medicine’s Centre for Health and Wellbeing. The WHO Quality of Life - Brief was used to assess the morbidity patterns and QOL of elderly individuals.

Observations: The gender-wise differences in each category revealed lower scores among males than females, but the difference was not statistically significant. The physical domain had a higher mean score (50.5 ± 15.5) than the psychological, social, and environmental domains. Type 2 diabetes, musculoskeletal disorders, and genitourinary disorders were the most prevalent morbidities, affecting 50% of the elderly population.

Conclusion: The relationship between multimorbidity and QOL in the elderly emphasizes the significant impact of multiple health conditions on overall wellbeing, leading to a poorer QOL. These findings highlight the necessity of targeted interventions that address the functional, social, and psychological aspects of medical management and care.

## Introduction

Old age is unavoidable, but it can be delayed and managed in good health by researching the causes of low quality of life (QOL) and addressing them through medical intervention or counseling. Both objective and subjective aspects are frequently used to describe QOL. Most senior citizens base their favorable assessments of their QOL on social interactions, independence, health, material status, and social comparisons. Resilience and adaptability may contribute to maintaining a high standard of living. While there are no cultural differences in the subjective aspects of QOL, variations exist in the objective aspects [1].

In the areas of autonomy, past, present, and future activities, social involvement, and closeness, the QOL of older adults was higher in family settings (60.62, 70.62, 66.14, and 58.43) than in old age homes (51.35, 62.91, 59.47, and 41.16; p<0.05) [2].

Among the prevalent morbidities observed in the research population, 42.8% experienced joint discomfort, 32.8% had cataracts, 22.4% had hypertension, 17.2% had diabetes mellitus, and 12.4% had dental issues. According to the QOL profile scoring, no elderly participants had a low QOL, while 56% fell into the "good" category and 50.8% into the "excellent" category. Males had significantly higher QOL scores than females across four distinct areas. Those married and living with their spouse had superior physical, environmental, and psychological conditions [3-5].

The psychological domain had the lowest mean QOL score (44.29 ± 11.50), followed by environmental health (51.64 ± 10.11) and social health (67.32 ± 15.30). Participants under 70 had higher scores in the physical health domain. Hindus, individuals from nuclear families, and those from higher socioeconomic classes exhibited better psychological health, while businessmen, men, and illiterate individuals had stronger social relationships. Those with higher socioeconomic status, business owners, and individuals with elementary education had substantially higher environmental domain scores [6-8].

In light of these findings, this study was designed to explore the relationship between the QOL of the elderly and various influencing factors. The primary objective was to analyze morbidity and QOL patterns using the WHO standardized scale.

## Materials and methods

An observational study was conducted among elderly individuals aged 60 years and above who visited the Department of Community Medicine’s Centre for Health and Wellbeing for a general health screening. The study included older adults aged 60 and above who participated in the Centre for Health and Wellbeing’s general screening program. However, it excluded those enrolled in the program but declined to participate. Participants were selected using a simple random sampling technique and were interviewed using a structured proforma after enrollment.

The Institutional Ethics Committee of BLDE (Deemed to be University) approved the study (approval number: BLDE(DU)/IEC/794/2022-23), and informed verbal consent was secured from the elderly patients at the Centre for Health and Wellbeing, which is housed in a tertiary care teaching hospital. Data collection was conducted between January 2023 and December 2023.

Sample size

A sample size of at least 100 elderly individuals enabled the study to assess the QOL with a margin of error of ±10%, a 95% confidence level, and an estimated lifetime prevalence of poor QOL of 50% among the elderly. Using the formula

$$n = \frac{Z^2 p (1 - p)}{d^2}$$

 where Z is the Z-statistic at a 5% significance level, and d represents the margin of error, a sample size of 100 was determined.

Study tool

There were 26 general and detailed questions on the WHO Quality of Life - Brief (WHOQOL-BREF). The first portion comprised two questions concerning general health status and overall QOL. The four health domains, physical, psychological, social, and environmental, were represented by 24 items. Seven variables were used to assess the physical health domain: pain, reliance on medical assistance, energy, mobility, sleep and rest, activities of daily living, and work capacity. Six items, positive feeling, personal belief, focus, body image, self-esteem, and negative feeling, were used to evaluate psychological wellness. Three questions about sexual life, social support, and personal relationships were answered regarding the dimension of social relationships. The results for each domain were converted to reflect a range of 4 to 20, with higher scores indicating a higher QOL. The WHOQOL-BREF had no overall score; instead, the total of the individual items in each domain determined the score.

Statistical analysis

A descriptive summary of each attribute will be provided. The summary statistics of the sample, including the mean and standard deviation (SD), were applied to continuous variables. Numbers and percentages were used to summarize categorical data. The data were analyzed using the Chi-square test for correlation and the t-test to compare the means.

## Results

One hundred fifteen elderly individuals (n=115) were enrolled in the study; 67 (58%) were female, and 48 (42%) were male. They belonged to the modified B.G. Prasad's classification of socioeconomic classes III (57 individuals, 50%) and IV (43 individuals, 38%).

According to Table 1, a greater number of participants (n=93, 81%) were in the age group of 60 to 70 years, married (n=72, 63%), educated at varying levels (n=101, 88%), not employed or retired (n=103, 90%), free from addictions such as alcohol or tobacco use (n=21, 18%), and had a BMI within the normal range (n=78, 68%). Except for habits, the frequency distribution of the two sets of variables was not found to be statistically significant. This could be due to cultural differences, as Indian women typically do not consume alcohol or smoke.

**Table 1:** Sociodemographic distribution of participants (n=115)

Variables		Male (n=48)		Female (n=67)		Chi-square value (p-value)
		No	%	No	%	
Age (years)	60-65 years	16	33%	32	48%	4.139 (0.246)
	65-70 years	19	40%	26	39%	
	70-75 years	09	19%	06	09%	
	>75 years	04	08%	03	04%	
Marital status	Married	24	50%	48	72%	5.751 (0.056)
	Unmarried	02	04%	01	01%	
	Widow/er	22	46%	18	27%	
Education	Illiterate	06	13%	08	12%	4.799 (0.308)
	Primary	02	04%	11	16%	
	Secondary	13	27%	17	25%	
	Pre-university	15	31%	20	30%	
	Graduate and above	12	25%	11	16%	
Occupation	Retired and not working	28	58%	39	58%	1.828 (0.400)
	Not working	13	27%	23	34%	
	Social work	07	15%	05	07%	
Socioeconomic class	Class II	09	19%	05	07%	3.332 (0.189)
	Class III	22	46%	35	52%	
	Class IV	17	35%	27	40%	
Habits	Alcohol and/or tobacco use	19	40%	02	03%	25.09 (0.0001)
	Nil	29	60%	65	97%	
BMI	Underweight	06	13%	09	13%	0.159 (0.923)
	Normal	32	67%	46	69%	
	Overweight/obese	10	21%	12	18%	

**Figure 1:**
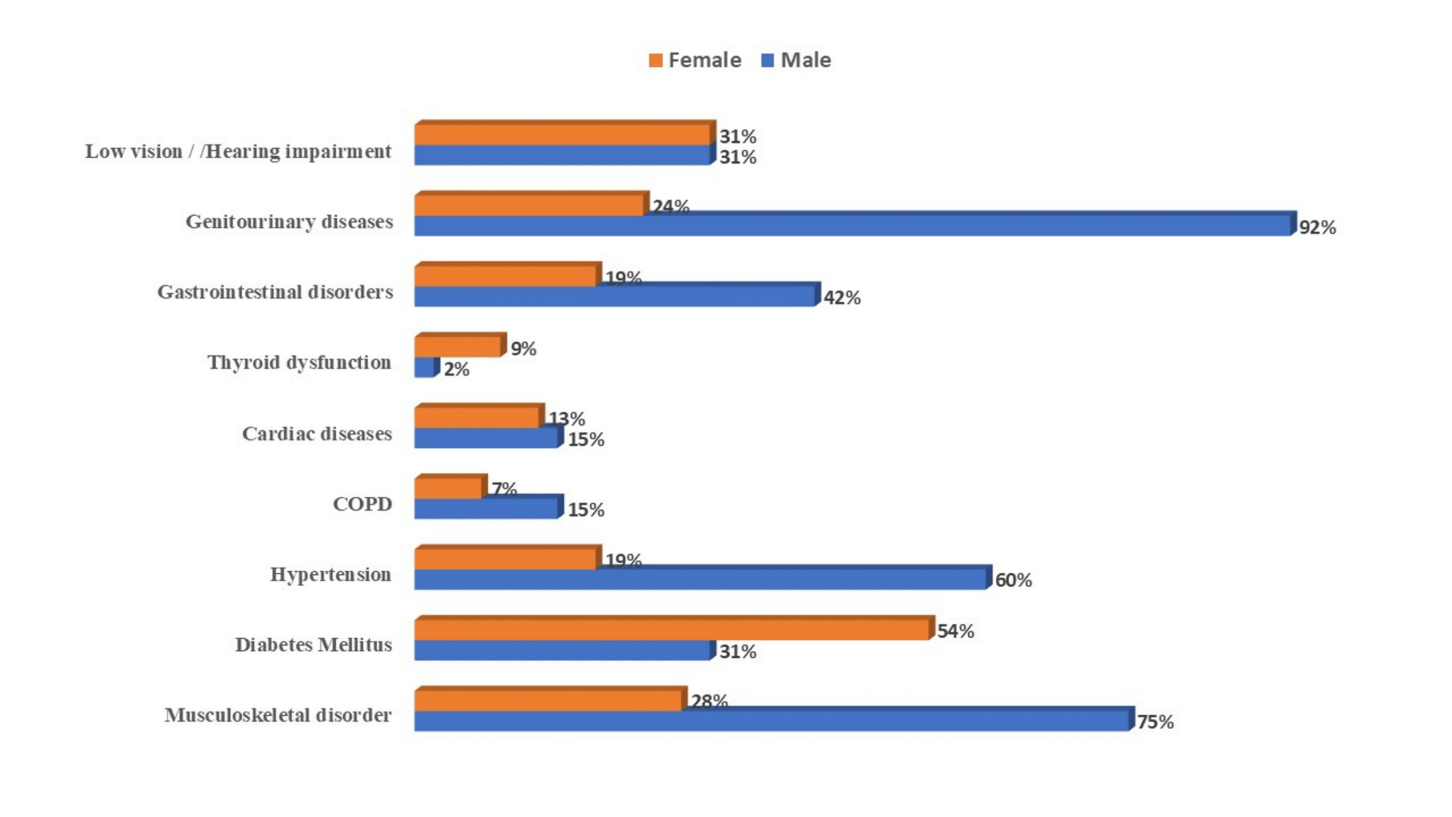
Gender-wise distribution of multimorbidity among old age individuals (N=115) COPD: chronic obstructive pulmonary disease

As shown in Figure 1, the majority of patients had genitourinary diseases (n=60, 52%), musculoskeletal disorders (n=55, 48%), and type-2 diabetes mellitus (n=51, 44%). On the other hand, only (n=7, 6%) of members had thyroid problems, (n=13, 11%) had chronic obstructive pulmonary disease (COPD), and (n=16, 14%) had heart disease.

Table 2 shows the distribution of the mean scores for the four dimensions of the WHO QOL for older adults. It indicates that the physical domain had a higher mean score (50.5 ± 15.5) than the social, psychological, and environmental domains. In contrast, the gender-based differences across each domain showed lower scores for males than females, though these differences were insignificant. The ANOVA test revealed no significant correlation between the mean scores across the various dimensions of QOL.

**Table 2:** Distribution based on QOL scores of old age individuals (N=115) QOL: quality of life, ANOVA: analysis of variance

QOL domain (0 to 100)	Male (n=48)	Female (n=67)	Total (n-115)	t-test (p-value)
Physical domain	49.9 ± 15.5	50.2 ± 15.4	50.5 ± 15.5	0.239 (0.811)
Psychological domain	47.0 ± 15.4	49.4 ± 15.6	49.2 ± 15.5	0.136 (0.891)
Social relationship domain	49.32 ± 16.9	49.4 ± 16.0	49.4 ± 16.5	0.038 (0.969)
Environmental domain	48.9 ± 15.4	49.5 ± 14.9	49.3 ± 15.1	0.2100 (0.834)
ANOVA	p=0.818	p=0.991	p = 0.9750	-

Table 3 shows that the mean QOL score was significantly lower for older female participants who were unmarried or widowed, retired, not employed, did not smoke or drink, and had no associated morbidities. In contrast, the mean score for all age groups was low but not statistically significant for those without education and those with lower socioeconomic status.

**Table 3:** Distribution of mean scores of QOL according to sociodemographic variables QOL: quality of life, SD: standard deviation

Variables		Mean score (n=115)		Unpaired t-test (p-value)
		Mean	SD	
Age (years)	60 to 70 years (n=100)	51.26	9.67	1.560 (0.121)
	>70 years (n=15)	47.03	10.62	
Gender	Male	55.4	9.9	3.367 (0.0001)
	Female	49.6	8.6	
Education	Illiterate (n=14)	48.61	10.42	0.963 (0.33)
	Literate (n=101)	51.45	9.67	
Marital status	Married (n=72)	53.09	8.32	5.181 (0.001)
	Not or lost (n=43)	43.87	10.6	
Occupation	Retired and not working (n=67)	46.38	11.70	2.301 (0.02)
	Working (n=43)	51.22	9.11	
Socioeconomic class	Class II and below (n=14)	50.48	10.73	0.456 (0.649)
	Class III and above (n=101)	49.19	9.81	
Habits	Present (n=21)	50.20	10.23	4.357 (0.0001)
	Absent (n=94)	42.43	6.62	
Associated morbidity	Present (n=60)	45.63	11.29	4.386 (0.0001)
	Absent (n=55)	51.98	8.83	

## Discussion

The present study shows the mean scores for four dimensions of the WHO QOL in old age. It reveals that the physical domain had a higher mean score (50.5 ± 15.5) than the social, psychological, and environmental domains. In contrast, the gender-wise difference between each domain showed lower scores among males than females, though this difference was insignificant. The majority of patients had genitourinary diseases (52%), musculoskeletal disorders (48%), and type-2 diabetes mellitus (44%). On the other hand, only 6% of the members had thyroid problems, 11% had COPD, and 14% had heart disease.

In their cross-sectional observational study of 295 elderly adults, Mishra et al. discovered that diabetes mellitus was the most common comorbidity, followed by hypertension. About 36% of them had two or more conditions, and women were more likely than men to have up to five chronic illnesses [9].

According to Ahmed et al., multimorbidity among Nigerian elderly individuals ranges from 27% to 74%, with cardiovascular conditions most frequently coexisting with metabolic and/or musculoskeletal disorders. There was a correlation between multimorbidity and age. In addition, factors such as women's gender, low educational attainment, economic hardship, joblessness, hospitalization, and emergency care were associated with a higher likelihood of multimorbidity [10].

According to Khanam et al., the prevalence of multimorbidity among the elderly was 53.8%, and it was significantly higher among women, those with less education, and those who were single or unmarried. Using logistic regression analysis, it was discovered that female sex and low economic position were separately linked to a higher odds ratio of multimorbidity. The results of the study aid in the planning of primary care for older patients in rural locations with many medical conditions [11].

According to research by Mujica-Mota et al., elderly patients who suffer from neurological conditions, arthritis, multi-morbid diabetes, or chronic mental health disorders experience a substantially lower QOL than those who do not. Integrated cognitive and physical health services are necessary for these individuals [12].

In their study, Kshatri et al. discovered that the prevalence of multimorbidity was 48.8%, with dyads accounting for 25% and triads for 15.2%. Males had a higher rate of multimorbidity (47.4%) than females (50.4%) [13].

Sum et al. claim that several non-communicable diseases impact QOL and healthcare utilization. To reduce adverse effects on QOL and effectively handle the demands of healthcare use, the healthcare system should consider the needs of older patients with various disorders [14].

Barca et al. conducted linear regression analyses and discovered that the following factors were linked to a lower QOL: a major depression diagnosis, a lower MMSE score, female gender, and decreased function in daily living activities [15].

Strengths and limitations of the study

Assessing the QOL among older adults helps to understand their unique challenges, such as chronic illnesses, disability, or other emotional and social impairments. Such studies are essential for planning healthcare practices and interventions to improve the wellbeing of elderly individuals. The study was conducted at the Centre for Health and Wellbeing with voluntary participants, and the findings cannot be generalized to the community as a whole.

## Conclusions

The current research on older adults’ QOL and its relationship to multimorbidity and other factors highlights the significant impact of these factors on overall QOL. The results emphasize the need for targeted therapies that address medical, social, functional, and psychological issues. When developing customized healthcare plans for elderly patients, it is crucial to identify the intricate interactions between factors such as age, gender, marital status, occupation, socioeconomic status, and multimorbidity.

## Appendices

**Table 4:** Proforma for data collection

Age (years)					
Gender			Male/female		
Education			Illiterate/literate		
Occupation			Retired and not working/not working/social work		
Socioeconomic status (according to the modified B.G. Prasad's classification			Class I/class II/class III/class IV/class V		
Morbidity details (last 6 months)			Mention		
History of hospitalization (in last 6 months)					
WHO quality-of-life questionnaires: the following questions ask about how much you have experienced certain things in the last two weeks					
	Not at all	A little	A moderate amount	Very much	An extreme amount
To what extent do impairments to your senses (e.g., hearing, vision, taste, smell, touch) affect your daily life?	1	2	3	4	5
To what extent does loss of, e.g., hearing, vision, taste, smell, or touch affect your ability to participate in activities?	1	2	3	4	5
How much freedom do you have to make your own decisions?	1	2	3	4	5
To what extent do you feel in control of your future?	1	2	3	4	5
How much do you feel that the people around you respect your freedom?	1	2	3	4	5
How concerned are you about how you will die?	1	2	3	4	5
How much are you afraid of not being able to control your death?	1	2	3	4	5
How scared are you of dying?	1	2	3	4	5
How much do you fear being in pain before you die?	1	2	3	4	5
The following questions ask about how completely you experienced or were able to do certain things in the last two weeks					
To what extent do problems with your sensory functioning (e.g., hearing, vision, taste, smell, touch) affect your daily life?	1	2	3	4	5
To what extent can you do things you’d like to do?	1	2	3	4	5
To what extent are you satisfied with the opportunities you have to continue achieving in life?	1	2	3	4	5
How much do you feel you have received the recognition you deserve?	1	2	3	4	5
To what extent do you feel you have enough to do daily?	1	2	3	4	5
The following questions ask you to say how satisfied, happy, or good you have felt about various aspects of your life over the last two weeks					
	Very dissatisfied	dissatisfied	Neither satisfied nor dissatisfied	Satisfied	Very satisfied
How satisfied are you with what you have achieved in life?	1	2	3	4	5
How satisfied are you with the way you use your time?	1	2	3	4	5
How satisfied are you with your level of activity?	1	2	3	4	5
How satisfied are you with your opportunity to participate in community activities?	1	2	3	4	5
	Very unhappy	Unhappy	Neither happy nor unhappy	Happy	Very happy
How happy are you with the things you can look forward to?	1	2	3	4	5
	Very poor	Poor	Neither poor nor good	Good	Very good
How would you rate your sensory functioning (e.g., hearing, vision, taste, smell, touch)?	1	2	3	4	5
The following question refers to any intimate relationships that you may have. Please consider these questions regarding a close partner or other close person with whom you can share intimacy more than with any other person in your life					
	Not at all	A little	A moderate amount	Very much	An extreme amount
To what extent do you feel a sense of companionship in your life?	1	2	3	4	5
To what extent do you experience love in your life?	1	2	3	4	5
	Not at all	A little	Moderately	Mostly	Completely
To what extent do you have opportunities to love?	1	2	3	4	5
To what extent do you have opportunities to be loved?	1	2	3	4	5
